# A new rapid and sensitive method for detecting chicken infectious anemia virus

**DOI:** 10.3389/fmicb.2022.994651

**Published:** 2022-09-29

**Authors:** Xiuhong Wu, Jie Kong, Ziqi Yao, Hejing Sun, Yuanjia Liu, Zhiqiang Wu, Jiajia Liu, Hao Zhang, Haohua Huang, Jin Wang, Mengjun Chen, Yichen Zeng, Yinpeng Huang, Feng Chen, Qingmei Xie, Xinheng Zhang

**Affiliations:** ^1^Heyuan Branch, Guangdong Provincial Laboratory of Lingnan Modern Agricultural Science and Technology, College of Animal Science, South China Agricultural University, Guangzhou, China; ^2^Guangdong Engineering Research Center for Vector Vaccine of Animal Virus, Guangzhou, China; ^3^South China Collaborative Innovation Center for Poultry Disease Control and Product Safety, Guangzhou, China; ^4^Department of Veterinary Medicine, College of Agricultural Sciences, Guangdong Ocean University, Zhanjiang, China; ^5^Wen’s Group Academy, Wen’s Foodstuffs Group Co., Ltd, Yunfu, Xinxing, China

**Keywords:** chicken infectious anemia virus, VP2, real-time recombinase-aided amplification assay, detection, real-time qPCR

## Abstract

Since the chicken infectious anemia virus (CIAV) was discovered in 1979, which has been reported as an economically significant and immunosuppressive poultry disease in the world. A novel clinical detection method for the prevention and control of CIAV in the poultry sector is urgently needed. Here, we established a real-time recombinase-aided amplification assay (RAA) for CIAV on-site with a rapid, highly sensitive, strongly specific, low-cost, and simple operational molecular diagnosis detection method. The primers and probe were developed using the CIAV VP2 gene sequence, which has a 117-bp specific band. This assay, which could be carried out at 41°C and completed in 30 min without cross-reactivity with other viruses, had the lowest detection limit of 10 copies of CIAV DNA molecules per reaction. Furthermore, the kappa value of this assay was 0.947, the sensitivity was 93.33%, and the specificity was 100% when compared to the real-time quantitative polymerase chain reaction assay (real-time qPCR). These results indicate that using a real-time RAA assay to detect CIAV on-site could be beneficial. In the future, the real-time RAA test may be a regular assay for the prevention and control of CIAV, as well as help the reduction of economic losses in the poultry business.

## Introduction

Chicken infectious anemia virus (CIAV) is an immunosuppressive virus, and its infection could cause aggravation of co-infections. CIAV is the only member of the genus gyrovirus of the family Anelloviridae with 20-dihedral symmetry and no envelope (Goryo et al., 1985; Natesan et al., 2006), and almost all of its ssDNA nucleic acids are 2,298 nt in length (Ducatez et al., 2006). CIAV contains three overlapped ORFs, encoding three distinct viral proteins: major capsid and immunogenic protein (VP1, 51.6 kDa), non-structural scaffold protein (VP2, 24.0 kDa), and apoptin (VP3, 13.6 kDa) (Noteborn et al., 1991; Shao et al., 2021). Chickens are the host of CIAV, and all ages are susceptible, mainly affecting chickens of 2–3 weeks. CIAV invades bone marrow hematopoietic tissue and thymus, leading to bone marrow chlorosis and thymus atrophy, and clinical symptoms are aplastic anemia and systemic lymphoid tissue atrophy (Yuasa et al., 1976). CIAV can be transmitted vertically or horizontally, resulting in widely spread around the world, which is one of the major diseases in the poultry industry and causes huge economic losses (Goryo et al., 1985; Abdel-Mawgod et al., 2018).

Since the discovery of CIAV was first reported in young broilers in 1996 in China, it has been widespread in different provinces and has caused huge economic losses in the poultry industry (Zhou et al., 1996). A seroprevalence report on CIAV in the 1990s showed that its prevalence was about 42% in farms in five provinces of China (Zhou et al., 1996). In the live poultry market in southeastern China live poultry market, the prevalence of CIAV was as high as 87% during 2004–2005 (Ducatez et al., 2008). Furthermore, the investigation of 12 provinces in China from 2014 to 2015 showed that the CIAV positivity rate was 13.30% (Yao et al., 2019). Recently, a survey showed that 91 of 277 chickens collected in a molecular epidemiological survey in Guangdong, China from 2016 to 2017 were positive for CIAV (Tan et al., 2020). Moreover, a survey showed that the CIAV positive rate was 47.58% by detecting tissue samples of 330 in 11 provinces in northern Vietnam in 2019 (Van Dong et al., 2019).

After infection with CIAV, the immune system of the body was suppressed, which was easy to cause secondary infection or complications, thus making prevention and control more difficult and expanding indirect economic losses (Hagood et al., 2000). The previous report showed that the Newcastle disease virus attenuated vaccine was detected CIAV and fowl adenovirus (FAdV) (Brown Jordan et al., 2019; Su et al., 2019). Chickens inoculated with contaminated CIAV vaccines became a subclinical viral infection that expanded the area of infection (Li et al., 2017). In addition, cross-infection made prevention and control more difficult, and should be brought to the attention of the stock farmers.

Nowadays, we use the methods of pathogen isolation and molecular biological detection for CIAV in the laboratory (Miller et al., 2003; Song et al., 2018; Kannaki et al., 2021), and serological tests such as enzyme-linked immunosorbent assay (ELISA) are used in clinical (Bhatt et al., 2011; Kabir et al., 2021). However, pathogen isolation and molecular biological detection require skillful personnel and expensive equipment. The target of ELISA was antibodies, which was limited by the time and concentration of antibody production, as someone cannot accurately detect it. Thus, a low-cost, high-sensitivity, specific, portable, and simple operable method is imperative to detect CIAV on site.

The real-time recombinase-aided amplification assay (RAA) method is a new molecular biology technique combining RAA with a portable fluorescence detection instrument to realize on-site detection (Wu et al., 2022a). The portable instrument has a built-in lithium battery, which can be used without plugging in grassroots farms with poor environmental conditions. The RAA method has been applied in food safety testing, environmental monitoring, aquatic disease detection, and animal and plant quarantine (Wang et al., 2019, 2021; Deng et al., 2021; He et al., 2021). They reported that RAA was applied in the detection of the Novel Coronavirus Disease 2019 (COVID-19) (Xue et al., 2020; Wang et al., 2020a; Shen et al., 2021). Previously, we established the application of real-time RT-RAA in avian leukosis virus subgroup J (ALV-J) and porcine epidemic diarrhea virus (PEDV) detection (Wu et al., 2022a,b). However, until now, there has been no report on CIAV detection by real-time RAA.

The RAA principle specifies that the recombinase and primer are tightly mixed at 35–40°C to form a polymer. With the help of single-stranded DNA binding protein and DNA polymerase, the primers search for complementary sequences on the template, and a new complementary DNA strand is formed (Chen et al., 2021). It is stable when the nucleic acid exonuclease is not activated, but when the probe begins to bind to the target DNA site, the exonuclease becomes active. When the nucleic acid exonuclease recognizes tetrahydrofuran (THF) on the probe, it cleaves both the reporter group and the burst group, releasing the reporter group. A blocker was removed as the probe ascended to the 3′ end, and the probe continued to amplify until the 5′ end. As a result, real-time fluorescence signals were gathered and plotted.

In this study, we used the VP2 gene sequence as the target for real-time RAA detection because it was the most conserved in CIAV, and the expression level of VP2 was higher than that of VP1 after infection in 12 h according to the previous reports (Douglas et al., 1995; Cheng et al., 2012; Lai et al., 2017).

## Materials and methods

### Virus and clinical specimens

The Marek’s Disease Virus (MDV, GenBank: L37202) vaccine was purchased from Harbin Pharmaceutical Group Bio-vaccine Co., Ltd. (Harbin, China). Wen’s Foodstuff Group Co, Ltd. (Yunfu, China) contributed to the Avian Reticuloendothelial Hyperplasia Virus (REV, not uploaded). In our laboratory, we retained the H9N2 subtype Avian Influenza Virus (H9N2, GenBank: MN064851), Infections Bursal Disease Virus (IBDV, GenBank: AF416621), Chicken Infectious Anemia Virus (CIAV, GenBank: JX260426), Newcastle Disease Virus (NDV, GenBank: JF950510), Infectious Laryngotracheitis Virus (ILTV, GenBank: JX458823), Avian Leukemia Virus (ALV, GenBank: MT175600) and Infectious Bronchitis Virus (IBV, GenBank: KR605489).

Wen’s Foodstuff Group Co., Ltd. donated 42 clinical samples for this trial (Yunfu, China). Thymus tissue samples from chickens suspected of being infected with CIAV were found from clinical samples and stored at −80°C in an ultralow temperature freezer.

### Sample preparation

The nucleic acid of MDV, ALV, IBV, NDV, IBDV, H9N2, ILTV, REV, and CIAV (GenBank: JX260426) were extracted following the instructions of the AxyPrep Body Fluid Viral DNA/RNA Miniprep Kit (AP-MN-BF-VNA-250G, Axygen, New York, United States). The forty-two clinical samples were extracted using the steps specified by the HiPure Tissue DNA Mini Kit (D3121, Magen, Guangzhou, China). The nucleic acid was then kept in an ultralow-temperature freezer at a temperature of 80°C.

### Reagents and instruments

The HiPure Tissue DNA Mini Kit was purchased from Guangzhou Magen Bio & Tech Co., Ltd. (D3121, Guangzhou, China). The AxyPrep Body Fluid Viral DNA/RNA Miniprep Kit was produced by Corning Incorporated (AP-MN-BF-VNA-250G, New York, United States). The freeze-dried powder for the real-time fluorescent RAA reaction was produced by Nanning Zhuangbo Bio & Tech Co., Ltd. (ZBA12001, Nanning, China). The EZ-press qPCR Kit was produced by Suzhou Yingze Biomedical Technology Co., Ltd. (EZB-qRT-R2, Suzhou, China). The pMD™19-T Vector Cloning Kit was produced by Takara Biomedical Technology (Beijing) Co., Ltd. (6,013, Beijing, China). This study used a Bio-Rad Laboratories CFX96 real-time fluorescence quantitative PCR apparatus (Shanghai, China).

### Generation of plasmid standard

The CIAV VP2 gene was amplified by PCR (Table 1). The primers were designed based on GD-1-12 (JX260426) and the product can be amplified at 559 bp. Recovery of PCR amplification products and cloned into pMD®19-T vectors (TaKaRa, China). After extraction, the concentration of this plasmid was calculated using the formula (6.02 × 10^23^) × (ng/μL × 10^−9^) / (DNA length× 660), with the copy number of the CIAV VP2 gene being almost 2.92 × 10^10^ copies/μL.

**Table 1:** Real-time qPCR and PCR assays of primers sequences for CIAV.

Assay	Primer	Sequence (5′-3′)	Localization
PCR	PCR-F	CAGTGAATCGGCGCTTAGCCG	394–414
	PCR-R	GATACCGCTGTCTCCTCCGA	933–952
Real-time qPCR	qPCR-F	CGGACCATCAACGGTGTTCAGG	494–515
	qPCR-R	GCAGCCACACAGCGATAGAGTG	590–611

### Primers and probe

The nucleotide sequences of CIAV VP2 were downloaded from the database from the National Center for Biotechnology Information^1^ (GenBank: AF285882, AF311892, AF311900, AF390038, AY040631, DQ991394, JX260426, KC414026, KF224934, KF224937, KJ872513, KU050680, KY486138, KY486141, KY888890, KY888903, KY888907, KY888910, KY888928, MK358456, M55918, MK687564, MN299313, MT268199, and MT795930), and we used the MEGAX64 (Mega Limited, Auckland, New Zealand) to align multiple sequences. Moreover, the Megalign of DNASTAR (DNASTAR, Inc., Madison, United States) was used to analyze homology and find the best region for designing the real-time RAA’s primers and probes. We also designed primers for real-time qPCR on conserved genes of CIAV VP2. The primers and probes were synthesized by Sangon Biotech (Shanghai) Co., Ltd. (Shanghai, China).

### Standard curve for real-time qPCR

The prepared CIAV VP2 gene plasmid was serially folded, and 6 dilution gradients of 10^7^–10^2^ copies/μL were used to construct a standard curve for real-time qPCR. Each dilution gradient was taken 2 μl and repeated three times. The real-time qPCR reaction system was as follows: qPCR-F (10 μM) 1.0 μl, qPCR-R (10 μM) 1.0 μl (Table 1), ddH_2_O 6.0 μl, 2 x SYBR Green qPCR Master Mix 10.0 μl, templates 2 μl. The real-time qPCR reaction procedure was: 1 cycle of 95°C for 5 min; 40 cycles by 95°C for 10 s, 60°C for 30 s, and fluorescent signals were collected during the elongation step. The protocol of melting curves was produced by monitoring the real-time SYBR green signal from 65°C to 95°C, incrementing 0.5°C for 0.05 s.

### The real-time RAA reaction system

The system of the real-time RAA per reaction was a total volume of 50 μl, including RT-RAA freeze-dried powder 1 tube, PEG buffer 25 μl, ddH_2_O 12.9 μl, MgAc_2_ buffer 2.5 μl, RAA-F (10 μM) 2.0 μl, RAA-R (10 μM) 2.0 μl, RAA-P (10 μM) 0.6 μl, and templates 5 μl. The real-time RAA amplification protocol was 1 cycle of 41°C for 60 s; 40 cycles of 41°C for 30 s and fluorescence signals were collected. After the reaction finished, the positive control had a smooth amplification curve, and the Ct value was <39; the negative control has no amplification curve, or the Ct value was>39, showing the assay has valid results.

### RAA primers verification and screening

A total of 12 groups of real-time primers were F1R1, F1R2, F1R3, F1R4, F2R1, F2R2, F2R3, F1R4, F3R1, F3R2, F3R3, and F1R4 (Table 2), and the size of the amplified product of the primers was verified by PCR. Meanwhile, the sensitivity of 12 groups of primers, respectively, in the real-time RAA system was evaluated. Based on PCR and real-time RAA results, the greatest primers were chosen. The PCR system and procedure as follows: forward primer (10 μM) 1.0 μl, reverse primer (10 μM) 1.0 μl, ddH_2_O 6.0 μl, 2xEsTaq Master Mix 10.0 μl, templates 2 μl; 95°C for 3 min; 95°C for 30 s, 58°C for 30 s, 72°C for 30 s, 35 cycles; 72°C for 5 min. 1.5% agarose gel electrophoresis was used to analyze the PCR products.

**Table 2:** Real-time RAA assays of primers and probes for CIAV.

Primer/probe	Sequence (5′-3′)	Localization
F1	TTCACGGCCGTTGGAAACCCCTCACTG	527–553
F2	AAACCCCTCACTGCAGAGAGATCCGGATTG	541–570
F3	AACAAGTTCACGGCCGTTGGAAACCCC	521–547
R1	TTGTCCGCAGTTGCAGATCTTAGCGTGG	628–655
R2	TTCGAGGGAGGCTTGGGTTGATCGGTC	695–721
R3	AATTGTCCGCAGTTGCAGATCTTAGCGTG	629–657
R4	TTCGAGGGAGGCTTGGGTTGATCGGTCC	694–721
probe	ATCGCTGGAATTACAATCACTCTATCGCTG/i6FAMdT//THF//iBHQ1dT/GGCTGCGCGAATGCTC[C3-spacer]	573–621

FAM, 6-carboxyfluorescein; THF, tetrahydrofuran; BHQ, black hole quencher; C3-spacer, 3′ phosphate blocker.

### Sensitivity analysis of the real-time RAA assay

The CIAV VP2 standard plasmid was serially diluted 10 times, and six concentration gradients were utilized as templates: 10^5^ copies/μL, 10^4^ copies/μL, 10^3^ copies/μL, 10^2^ copies/μL, 10^1^ copies/μL, and 10^0^ copies/μL. The sensitivity assay was then analyzed by repeating it five times. To achieve an accurate evaluation of sensitivity, the different dilution series (10^4^–10^−1^ TCID50 CIAV DNA per reaction) were used as templates in both real-time qPCR and real-time RAA analyses five-time independently.

### Specificity analysis of the real-time RAA assay

For the real-time RAA, the CIAV nucleic acid was performed as a positive control, while ddH_2_O worked as a negative control. For specificity analysis of MDV, ALV, IBV, NDV, IBDV, H9N2, ILTV, and REV nucleic acids, 2 μl of templates were acquired and repeated three times.

### Clinical sample evaluation of the real-time RAA assay

Real-time qPCR and real-time RAA were utilized to detect nucleic acids from 42 clinical samples suspected of being infected with CIAV. We compared and analyzed the results of real-time qPCR and RAA to evaluate the real-time RT-RAA method developed in this study.

### Statistical analysis

Results of real-time RAA and real-time qPCR assays were measured with kappa and *p*-values at 95% reliability using IBM’s SPSS software.

## Results

### Designing the real-time RAA primers and probe of CIAV

**Figure 1:**
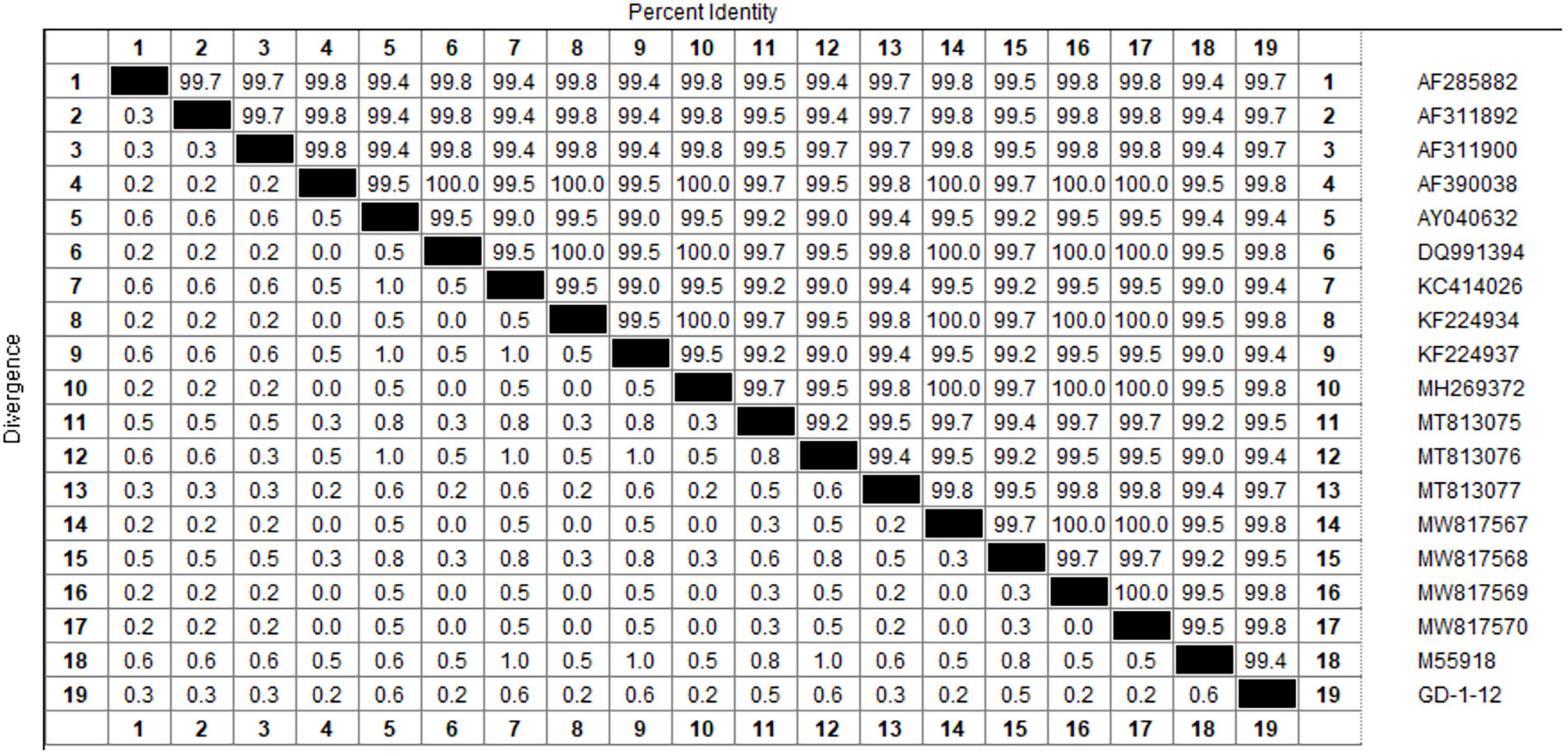
The multiple sequence alignment of the CIAV VP2 gene. Multiple sequence alignment using GD-1-12 as the reference strain revealed CIAV VP2 had greater homology than 99.4%. Both MEGAX64 and Megalign of DNASTAR used were default parameters. RAA, recombinase-aided amplification assay.

Based on the VP2 sequence of the GD-1-12 (JX260426) strain isolated in our laboratory, we performed multiple sequence alignment with the sequence published in the NCBI database using Megalign’s castal W method. The homology values of CIAV VP2 sequences were over 99.4%, indicating great conservatism, as shown in Figure 1. We designed primers and probes for CIAV real-time RAA on this gene fragment depending on the results of homology analysis, as shown in Table 2. The sequences of the CIAV VP2 had not intersected with other viruses, according to NCBI’s BLAST analysis of the amplified fragments of the primers and probe.

### Verification and screening of real-time RAA primers for CIAV

**Figure 2:**
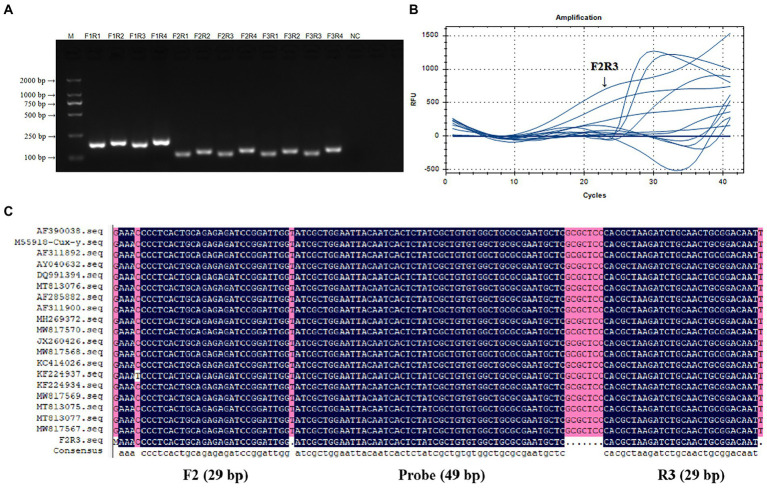
Verification and screening of real-time RAA Primers for CIAV. Twelve groups of real-time RAA primers for CIAV were F1R1, F1R2, F1R3, F1R4, F2R1, F2R2, F2R3, F1R4, F3R1, F3R2, F3R3, and F1R4 that verified by PCR amplification **(A)** and real-time RAA assay **(B)**. F2R3 had a higher amplification efficiency than the other primers in real-time RAA assay, with a 117 bp specific band size and no-primer-dimers in 1.5% gel electrophoresis. **(C)** A multiple sequence alignment of the F2R3 and probe sequences with the primers’ information amplification length. M, D 2000 Marker; N, negative control, i.e., ddH_2_O. RAA, recombinase-aided amplification assay; CIAV, Chicken Infectious Anemia Virus.

PCR products were performed by 1.5% gel electrophoresis to verify the size and specificity of real-time RAA primers. Figure 2A showed the results. The size of amplified fragments from 12 different primers both met predictions and did no-primer-dimers, which has good specificity for CIAV. Figure 2B showed the results of a real-time RAA process using the nucleic acid of CIAV as the template. Under the same template concentration, F2R3 had a higher amplification efficiency than the other primers. Figure 2C showed the result of multiple sequence alignment of F2R3 and probe sequences. F2R3 was chosen as the best primer for CIAV real-time RAA based on the above results.

### Standard curve of real-time qPCR for CIAV

**Figure 3:**
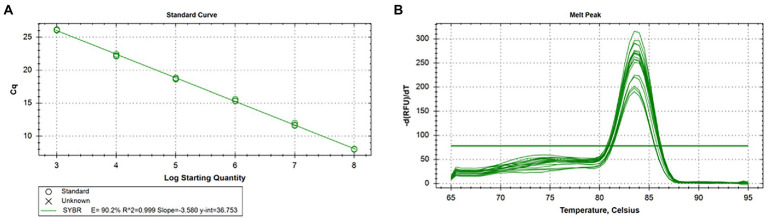
Standard curve and melting curve of real-time qPCR for CIAV. **(A)** The logarithmic function relationship between cycle threshold and plasmid copy number was y = −3.580x + 36.753, R^2^ = 0.999, and efficiency was 90.2%. **(B)** The melt temperature of real-time qPCR for CIAV was 83.5°C. CIAV, Chicken Infectious Anemia Virus; qPCR, quantitative polymerase chain reaction assay.

According to the results, the standard curve exhibits a good linear relationship with the correlation coefficient of R^2^ value was 0.999, the amplification efficiency was 90.2%, and the melt temperature was 83.5°C. The logarithmic function relationship between cycle threshold and plasmid copy number was y = −3.580x + 36.753 and was shown in Figures 3A,B.

### Sensitivity analysis of The real-time RAA assay for CIAV

**Figure 4:**
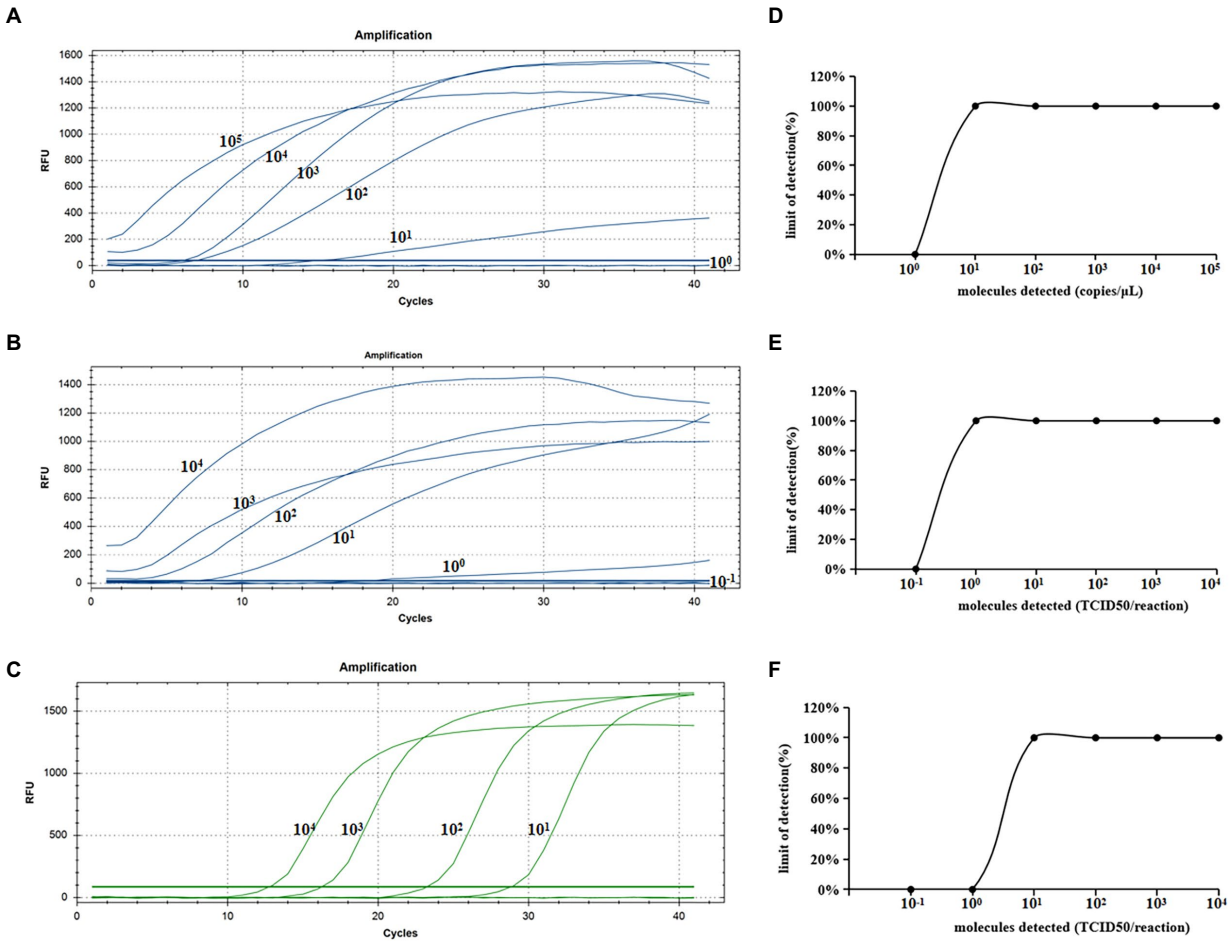
Sensitivity analysis of the real-time RAA and real-time qPCR assay for CIAV. **(A)**The standard plasmid of CIAV VP2 was detected at 10^5^, 10^4^, 10^3^, 10^2^, 10^1^, and 10^0^ copies/μL, repeated five, respectively. Ten copies of DNA can be detected per reaction of the real-time RAA assay. **(B)** The real-time RAA assay detected CIAV TCID50 10^−1^ to 10^4^ per reaction repeated five, respectively. **(C)** The real-time qPCR assay detected CIAV TCID50 10^−1^ to 10^4^ per reaction repeated five, respectively. **(D)** The data of five runs from plasmid standards by analyzing real-time RAA assay. **(E)** The data of five runs from CIAV DNA by analyzing real-time RAA assay. **(F)** The data of five runs from CIAV DNA by analyzing real-time qPCR assay. CIAV, Chicken Infectious Anemia Virus; qPCR, quantitative polymerase chain reaction assay; RAA, recombinase-aided amplification assay.

As shown in Figure 4, we chose a typical result from the five independent real-time RAA assays and real-time qPCR. Figures 4A–C showed that the real-time RAA in 10^1^ copies/μL of standard plasmid, and 10^0^ TCID50/reaction of CIAV, and the real-time qPCR 10^1^ TCID50/reaction had fluorescence signals among the five times results from the six concentrations gradients. Figures 4D–F showed probit regression analysis of the real-time RAA and qPCR, using SPSS software on data from the five runs of standard plasmid and CIAV DNA. As a result, the limited sensitivity of the real-time RAA we developed for CIAV was 10 copies of standard plasmid and 1 TCID50 of CIAV can be detected per reaction.

### Specificity analysis of the real-time RAA assay for CIAV

**Figure 5:**
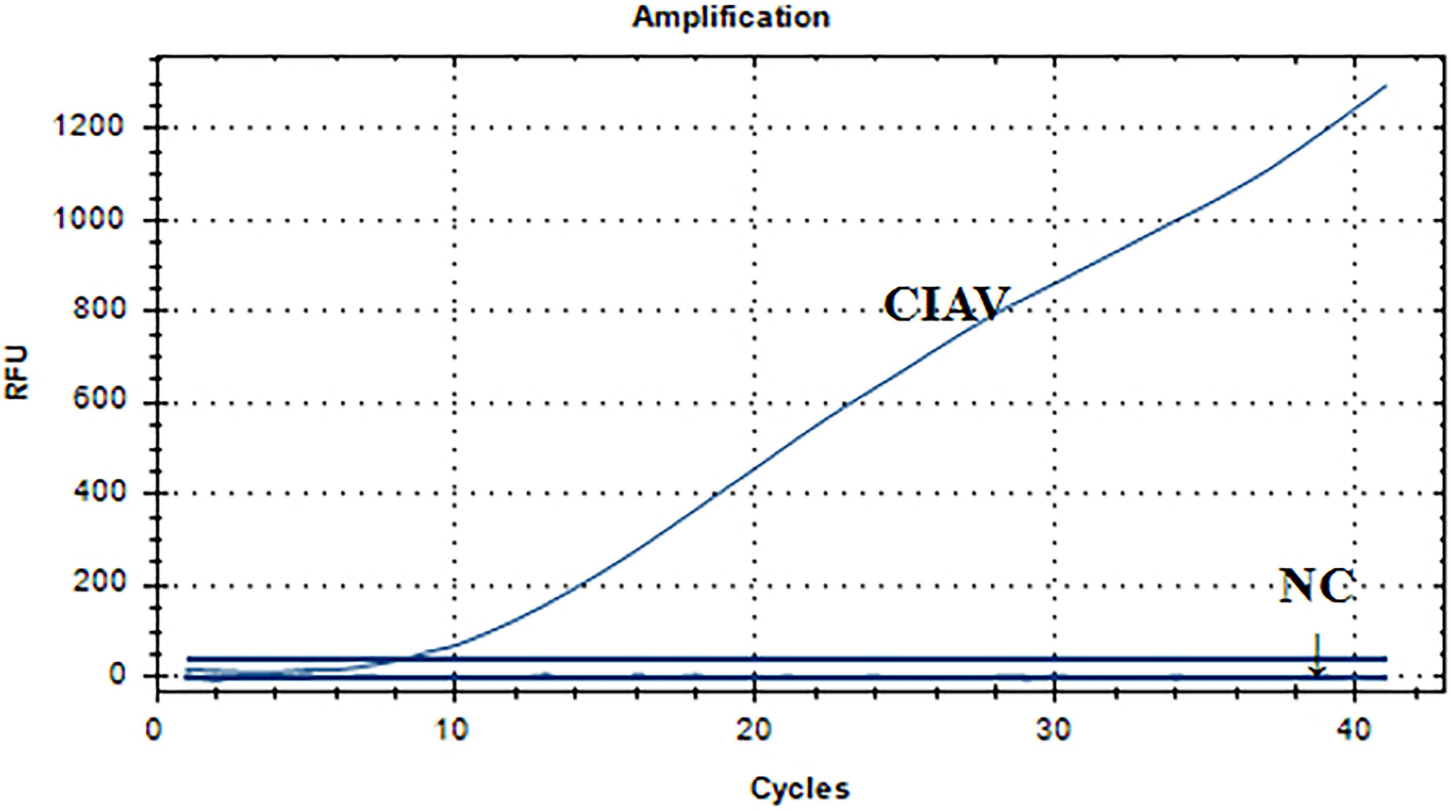
Specificity analysis of the real-time RAA assay for CIAV. The CIAV nucleic acid positive control had a single fluorescence signal curve. There was no fluorescence signal in the negative controls of ddH_2_O, H9N2, MDV, ALV, ILTV, IBV, NDV, REV, and IBDV nucleic acid. NC: negative control, i.e., ddH_2_O, H9N2, MDV, ALV, ILTV, IBV, NDV, REV, and IBDV nucleic acid. RAA, recombinase-aided amplification assay; MDV, Marek’s Disease Virus; CIAV, Chicken Infectious Anemia Virus; IBV, Infectious Bronchitis Virus; NDV, Newcastle Disease Virus; IBDV, Infections Bursal Disease Virus; H9N2, Avian Influenza Virus subgroup H9N2; ILTV, Infectious Laryngotracheitis Virus; REV, Avian Reticuloendotheliosis Virus; ALV, avian leukosis virus.

Figure 5 showed a representative result from the three independent real-time RAA assays. The CIAV nucleic acid positive control had a single fluorescence signal curve. There was no fluorescence signal in the negative controls of ddH_2_O, H9N2, MDV, ALV, ILTV, IBV, NDV, REV, and IBDV nucleic acid. As a result, we developed the real-time RAA method that could specifically identify CIAV while exhibiting no cross-reactivity with MDV, ALV, IBV, NDV, IBDV, H9N2, ILTV, or REV.

### Clinical sample evaluation of the real-time RAA assay for CIAV

Table 3 showed that of the nucleic acids of the 42 clinical samples, 14 nucleic acids were positive amplification by real-time qPCR and real-time RAA assays. However, one of the nucleic acids showed positive amplification by real-time qPCR but negative amplification by real-time RAA. Real-time qPCR and real-time RAA both have a sensitivity of 93.33%. None of the 27 nucleic acids had a fluorescence signal for real-time qPCR and real-time RAA assays, indicating negative amplification with a specificity of 100%. This assay’s kappa value of real-time RAA was 0.947 compared to the real-time qPCR assay, showing that the two methods were almost perfect.

**Table 3:** Comparison of the results of real-time RAA assay and qPCR method for clinical samples.

		Real-time qPCR		Performance characteristics		
		Positive	Negative	Sensitivity (%)	Specificity (%)	Kappa
Real-time RAA	Positive	14	0	93.33		0.947
	Negative	1	27		100	
	Total	15	27			

## Discussion

Researchers have developed many vaccines to prevent and control CIAV from an immunization perspective to reduce the significant production losses carried on by clinical and subclinical infections in commercial poultry because of the prevalence of CIAV in producing countries’ broilers (Barbour et al., 2002; Vaziry et al., 2011; Sawant et al., 2015). However, the CIAV antibody response remained low in most vaccinated chicks and did not persist until day 18 post-immunization, and the vaccine strain persisted in the thymus and spleen of young chicks, resulting in a low humoral immune response and inducing thymic lymphoid cell disorders (Vaziry et al., 2011). Persistent infection was more likely to result from this subclinical infection phenomenon. For example, the dual infection of CIAV and infectious bursal disease virus (IBDV) had no obvious clinical symptoms, but the detection of the thymus showed persistent infection of CIAV (Vaziry et al., 2013). The CIAV co-infection with FAdV had obvious inclusion of body hepatitis- hydropericardium syndrome (Su et al., 2019). The reality that CIAV can infect a different species complicates matters further and makes it more challenging to prevent and control (Zhang et al., 2014; Fang et al., 2017). Therefore, an effective, accurate, and simple-to-operate detection method is required to better monitor and prevent CIAV outbreaks while also reducing the poultry industry’s economic losses.

Researchers have developed a variety of detection methods for CIAV, each with its own set of benefits and drawbacks. As indicated in Table 4, we provide a brief overview of the different CIAV detection methods. The virus isolation period of CIAV is the longest, at least 14 days (Buscaglia et al., 1994). Before noticeable cytopathic alterations may be seen with a fluorescence microscope, MDCC-MSB1 cells must be infected with CIAV for more than 24 h, and there are issues for immunofluorescence assays (IFA) detect CIAV with non-specificity and insufficient sensitivity (Lucio et al., 1991; Buscaglia et al., 1994). Compared to other detection methods, virus isolation and IFA require skilled, high-quality personnel, and sterile negative pressure environments, both of which are more expensive. The most commonly detected CIAV with two methods, PCR and qPCR, with the assay taking between 108 and 137 min (Todd et al., 1992; Kaffashi et al., 2021). However, PCR requires variable temperature equipment and electrophoresis (Zhang et al., 2020). It is possible to obtain accurate results in real-time with qPCR, but it requires expensive equipment and partitioned reaction systems to reduce the false-positive rate (Yoo et al., 2021). LAMP needs four pairs of primers to detect CIAV at 63°C for 40 min, and the results can be visualized, making it convenient for clinical testing (Song et al., 2018). However, four pairs of primers with eight ssDNA in one tube are prone to form primer-dimers, increasing the LAMP detection of false-positive results (Wang et al., 2020b). In addition to being the easiest method of detection, the ELISA test also is commonly used in clinical to detect CIAV (Pallister et al., 1994; Tannock et al., 2003). Due to the low level of CIAV antibodies in the vaccinated chicks, no antibody can be detected. There are problems with non-specific binding and low sensitivity with an ELISA (Michalski et al., 1996).

**Table 4:** Different detection methods for CIAV.

Methods	Sensitivity	Instrument requirements	Assay time	On-site	References
Real-time RAA	10 copies	Portable fluorescence reporter	30 min	YES	This study
PCR	0.1 pg	PCR; Electrophoresis System; Transilluminator	137 min	NO	Todd et al., 1992
qPCR	21 copies	Fluorescence PCR	108 min	NO	Kaffashi et al., 2021
LAMP	50 copies	Portable fluorescence reporter	40 min	YES	Song et al., 2018
ELISA	–	ELIASA	135 min	YES	Li, 2021
IFA	–	Fluorescence microscope	24 h	NO	Lamichhane et al., 1992
Virus isolation	–	Laboratory of cell biology	At least 14 day	NO	Buscaglia et al., 1994

In this study, we developed the real-time RAA that can overcome the deficiencies of the above method in the clinical detection of CIAV. When combined with recombinase at a constant temperature (39–42°C), the real-time RAA primers only further amplify once it matches the template with the completed complementary sequence, thus reducing error tolerance and improving detection accuracy. We designed 12 groups of primers for the highly conserved VP2 gene to select the optimal primers and performed double screening by PCR and real-time RAA. We used the screened F2R3 primers to evaluate its specificity with MDV, ALV, IBV, NDV, IBDV, H9N2, ILTV, and REV, and the results showed that there was no amplification reaction with these viruses, which further proved that the primers we designed can specifically detect CIAV. Sensitivity demonstrated that real-time RAA could detect a limitation of 10 copies per reaction of standard plasmid, and 1 TCID50 per reaction of CIAV, and the detection results of six concentration gradients in five repeated experiments had a 100% coincidence rate, which also showed that our established real-time RAA had high sensitivity and reproducibility. In other words, real-time RAA can be used helpfully for clinical diagnosis, whether it is infected by CIAV in the clinical symptom or early stages. Noteworthy, the real-time RAA detection results of 42 clinical samples compared with qPCR, the performance characteristics of kappa value was 0.947, sensitivity was 93.33%, and specificity was 100%, indicating the accuracy of the results was consistent with qPCR. What is more superior is that real-time RAA can be completed within 30 min at 41°C and combined with a portable fluorescence detector to move detection in a complex environment to complete accurate detection leave out the laboratory.

The real-time RAA method for rapid detection of CIAV has the characteristics of simple operation, high sensitivity, strong specificity, and short detection time that can better prevent and control CIAV in clinical. The real-time RAA has excellent detection performance and can be well implemented in early diagnosis, long-term monitoring, clinical mobile detection, and cross-host transmission of CIAV.

## Conclusion

This study successfully established and applied a real-time RAA method to detect CIAV within 30 min at 41°C. In the poultry industry, real-time RT-RAA offers high performance in sensitivity, specificity, repeatability, and low cost, which could be key for preventing and controlling CIAV.

## Data availability statement

The raw data supporting the conclusions of this article will be made available by the authors, without undue reservation.

## Ethics statement

Institutional and National Guidelines for the use and care of laboratory animals were closely followed. The use of animals in this study was approved by the South China Agricultural University Committee for Animal Experiments (approval ID: SYXK2019-0136).

## Author contributions

XW conducted the experiments, analyzed the data, and wrote the manuscript. JK, ZY, HS, HZ, HH, JW, MC, YZ, and YH conducted the experiments. FC analyzed part of the data. YL, ZW, and JL checked and finalized the manuscript. QX and XZ designed the experiments, modified the manuscript, and supervised the whole work. All authors have read and approved the final manuscript.

## Funding

This work was supported by the Guangdong Basic and Applied Basic Research Foundation (2019A1515012006), the Key Research and Development Program of Guangdong Province (2020B020222001), the National Natural Science Foundation of China (Grant Nos. 31902252 and 31972659), and the Chief expert Project of Agricultural Industry Technology System in Guangdong Province (2019KJ128).

## Conflict of interest

JL and ZW were employed by Wen’s Foodstuffs Group Co., Ltd.

The remaining authors declare that the research was conducted in the absence of any commercial or financial relationships that could be construed as a potential conflict of interest.

## Publisher’s note

All claims expressed in this article are solely those of the authors and do not necessarily represent those of their affiliated organizations, or those of the publisher, the editors and the reviewers. Any product that may be evaluated in this article, or claim that may be made by its manufacturer, is not guaranteed or endorsed by the publisher.

## Footnotes

^1^ https://www.ncbi.nlm.nih.gov
